# Combined recurrent laryngeal nerve monitoring and one-lung ventilation using the EZ-Blocker and an electromyographic endotracheal tube

**DOI:** 10.1186/s13019-019-0927-6

**Published:** 2019-06-19

**Authors:** Andreas Moritz, Joachim Schmidt, Waldemar Schreiner, Torsten Birkholz, Horia Sirbu, Andrea Irouschek

**Affiliations:** 1 Department of Anesthesiology, University Hospital of Erlangen, Krankenhausstrasse 12, 91054 Erlangen, Germany; 2 Department of Thoracic Surgery, University Hospital of Erlangen, Krankenhausstrasse 12, 91054 Erlangen, Germany

**Keywords:** Thoracic surgery, EZ-Blocker, Recurrent laryngeal nerve, One-lung ventilation, Intraoperative monitoring

## Abstract

**Background:**

Intraoperative neuromuscular monitoring (IONM) is a widespread procedure to identify and protect the recurrent laryngeal nerve (RLN) during thyroid surgery. However, for left thoracic surgery with high risk of RLN injury, both reliable recurrent laryngeal nerve monitoring and one-lung ventilation could interfere.

**Methods:**

In this prospective study, a new method for IONM during one-lung ventilation combining RLN monitoring with an electromyographic (EMG) endotracheal tube (ETT) and lung separation using the EZ-Blocker (EZB) is described and its clinical feasibility and effectiveness were assessed.

**Results:**

A total of 14 patients undergoing left upper lobe surgery and left upper mediastinal lymph node dissection were enrolled. The EZB was introduced and positioned without any problems and sufficient lung collapse was achieved in all patients. No tracheobronchial injuries or immediate complications occurred. A stable EMG signal was present in all patients and no RLN palsy and no negative side effects of the NIM EMG ETT or the EZB were observed postoperatively.

**Conclusions:**

The described method is technically feasible, easy to apply and save. It provides both reliable IONM and independent lung separation for optimal surgical exposure. The combined use of the EZB and the NIM EMG ETT might reduce the risk for RLN palsy and impaired lung separation during left thoracic surgery with high risk for RLN injury.

## Background

Recurrent laryngeal nerve (RLN) injury is one of the most common complications of thyroid and parathyroid surgery. The incidence of transient and permanent RLN injury in thyroidectomy are 2–11% and 0.6–1.6%, respectively [1]. In thoracic surgery for lung cancer the incidence rate of RLN injury was reported to be ranging from 1.5% [2] to 31% [3] according to the surgical procedure. For different types of transthoracic surgery for esophageal cancer RLN injury ranges between 0 and 59% [4–6] and is associated with a significant morbidity, especially pulmonary complications [7]. It has been reported that intraoperative nerve monitoring (IONM) could reduce the incidence of total, transient and permanent RLN injury in patients undergoing thyroid surgery [1]. Therefore, IONM is frequently used in thyroid surgery as a useful tool for visual nerve identification. However, data regarding the effectiveness of nerve monitoring during thoracic surgical interventions are scarce [8]. In addition, IONM for thoracic surgical interventions still poses a significant challenge because of the need for one-lung ventilation during surgical exposure.

We implemented a novel approach to identify and preserve the RLN during lung separation using the EZ-Blocker (EZB; AnaesthetIQ, Rotterdam, The Netherlands) in combination with a single-lumen electromyographic (EMG) endotracheal tube (ETT) (NIM EMG Endotracheal Tube, Medtronic Xomed, Jacksonville, Florida, USA). As previous described, the EZB is an efficient, easy-to-use and safe airway device to allow single-lung ventilation and to provide optimal surgical exposure [9]. Therefore, in surgical procedures with necessity of lung separation and high risk for RLN injury the new method might be advantageous to provide both, reliable IONM and optimal surgical exposure.

The aim of the present study was to determine the clinical feasibility and effectiveness of the combined use of the EZB and the NIM EMG ETT for IONM during one-lung ventilation in patients undergoing left upper lobe surgery and left upper mediastinal lymph node dissection for cancer with high risk for RLN injury.

## Methods

The present prospective feasibility study was performed at the University Hospital of Erlangen and was evaluated and approved by the institutional ethics committee (reference number: 4590). Patient recruitment and data retrieval was according to the good clinical practice guidelines. All patients provided written informed consent. Following inclusion into the study, no further selection was performed.

The University Hospital of Erlangen provides the full spectrum of thoracic surgery. Anesthesia induction, maintaining and IONM were performed according to the standard operating procedures (SOPs) of the hospital, using a total intravenous anesthesia (TIVA). After neuromuscular block for intubation, neuromuscular transmission was monitored with acceleromyography at the adductor pollicis muscle (TOF-Watch SX, Organon Ireland Ltd., a division of Merck and Co. Inc., Swords, Co. Dublin, Ireland) to exclude a persistent neuromuscular block during IONM.

**Fig. 1:**
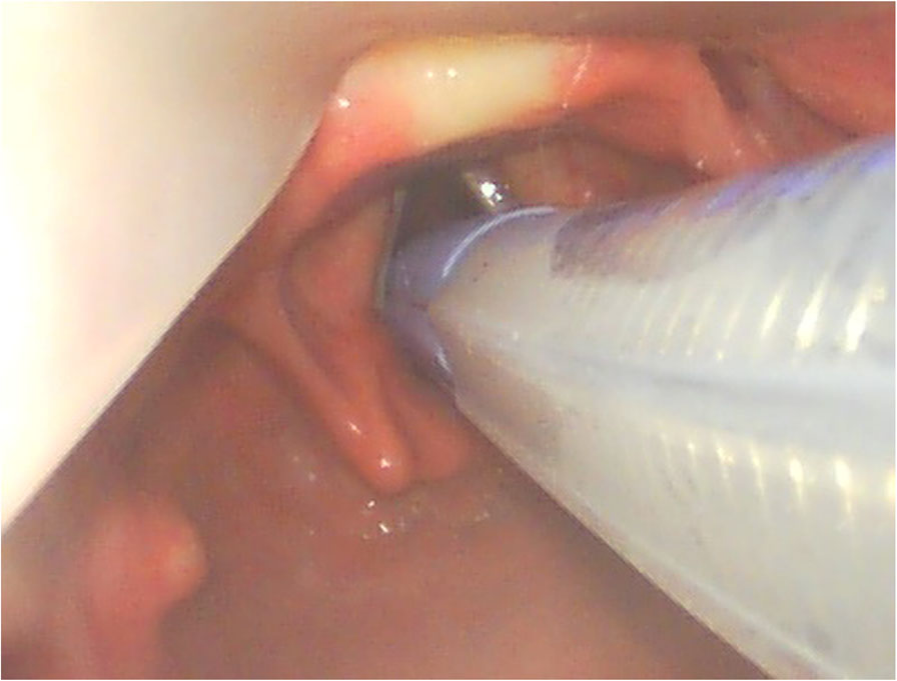
Videolaryngoscopic view of the NIM EMG ETT placed in the larynx with the middle of the exposed electrodes well in contact with the true vocal cords

**Fig. 2:**
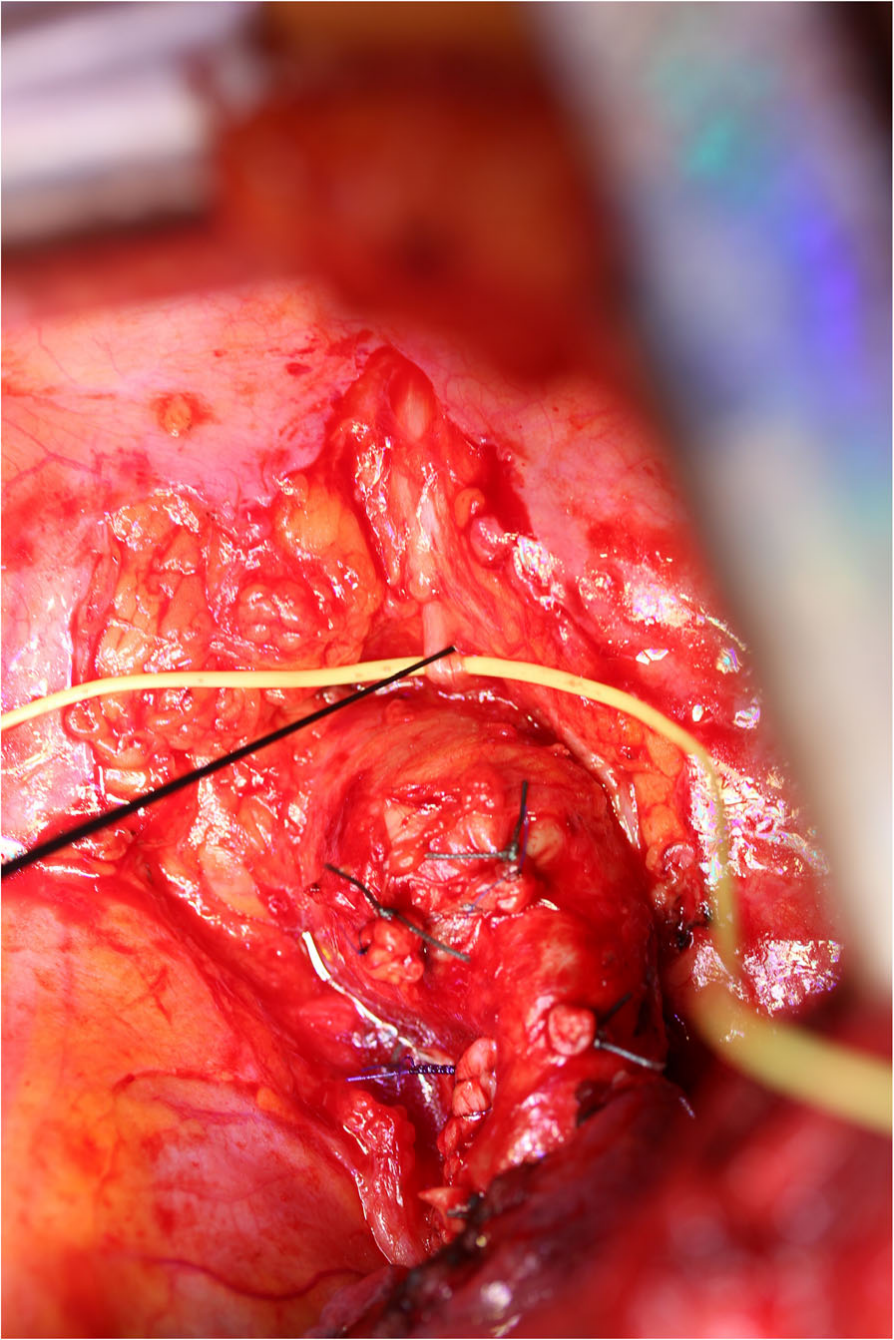
Electrical stimulation of the left RLN with a monopolar nerve stimulator probe during left upper lobe resection. The RLN is identified and encircled with a vascular loop. The left upper lobe pulmonary artery branches are ligated

All patients were intubated orotracheally with a NIM EMG ETT (Medtronic Xomed, Jacksonville, Florida, USA) (7 mm I.D. for female patients, 8 mm I.D. for male patients). The NIM EMG ETT is a flexible silicone elastomer single-lumen ETT with an inflatable cuff. The tube is fitted with four stainless steel wire electrodes (two pairs) which are embedded in the silicone of the main shaft of the ETT and exposed only for a short distance (28 mm), slightly superior to the cuff, for contacting the true vocal cords. Correct positioning of the surface electrodes is essential to enable a side related EMG signal monitoring. Therefore the NIM EMG ETT was placed with the middle of the exposed electrodes well in contact with the true vocal cords (Fig. 1) using video laryngoscopy (Glidescope, Verathon Medical, Rennerod, Germany or C-MAC Karl Storz, Tuttlingen, Germany). After the patient had been placed in right decubitus position, a grounding electrode and an anode electrode were placed in the subcutaneous tissue of the left shoulder. All electrodes were connected to the NIM-Response 3.0 monitor (Medtronic Xomed, Jacksonville, Florida, USA). Correct positioning of the laryngeal surface electrodes was confirmed by transcutaneous cervical suprathreshold vagal stimulation using an electrical nerve stimulator (NS 252, Fisher and Paykel Healthcare Electronics Ltd., Auckland, New Zealand). In addition, the NIM-Response 3.0 monitor was used to verify electrode impedance measures less than 5 KΩ. The event threshold was set at 100 μV and an activated evoked potential greater than 100 μV was considered as a positive EMG signal. A cutoff device (cylindrical clamp) was secured around the electrocautery wire to sense current passing through the wire with each electrocautery use. To identify and preserve the RLN during surgery, the course of the RLN was explored by using a monopolar stimulating electrode (Prass, Medtronic Xomed, Jacksonville, Florida, USA) (Fig. 2). The monopolar probe provides a more diffuse current spread and thus could facilitate mapping out the RLN when compared to bipolar nerve stimulator probe where the stimulation is localized at the point of contact [10]. The stimuli for mapping out were generated from the NIM-Response 3.0 monitor and the intensity was typically 1.0 mA for RLN nerve mapping. However, once the RLN is visualized, the stimulation current could be turned down. Stimulation of the RLN or vagal nerve results in an audible and visual EMG signal on the monitor screen. During surgery, a three-step procedure of IONM was used: To ensure that the EMG monitoring system is functioning correctly and that the RLN remains on a normal path, a sequential stimulation of the vagal nerve was performed before dissection. To preserve the RLN during surgery, the course of the RLN was labeled by using the monopolar probe. After the tumor and/or lymph nodes had been completely dissected and hemostasis had been completed, the integrity of the RLN was confirmed again by sequential stimulation of the vagal nerve.

**Fig. 3:**
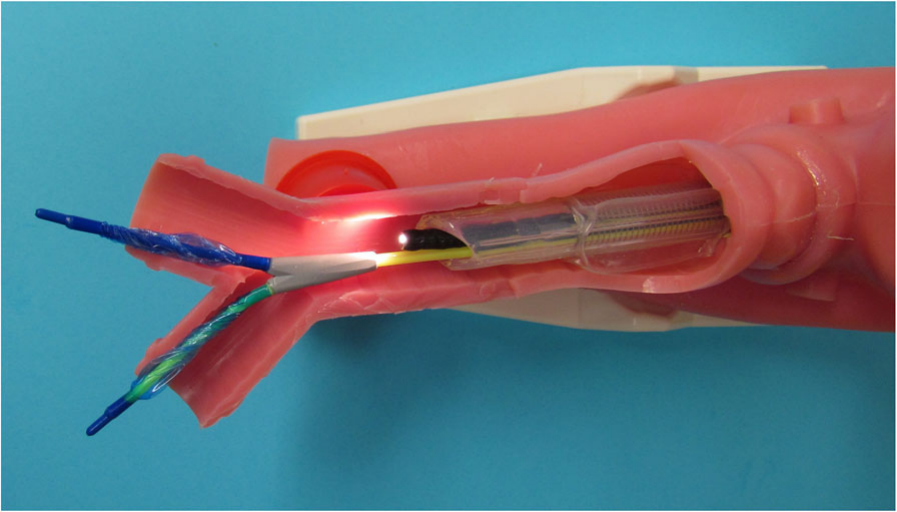
Close-up view of the EZB placed through a NIM EMG ETT in a manikin. The Y-shape of the distal portion facilitates the anchorage of the blocker to the carina. The two distal extensions are colored differently, both with an inflatable cuff and a central lumen

**Fig. 4:**
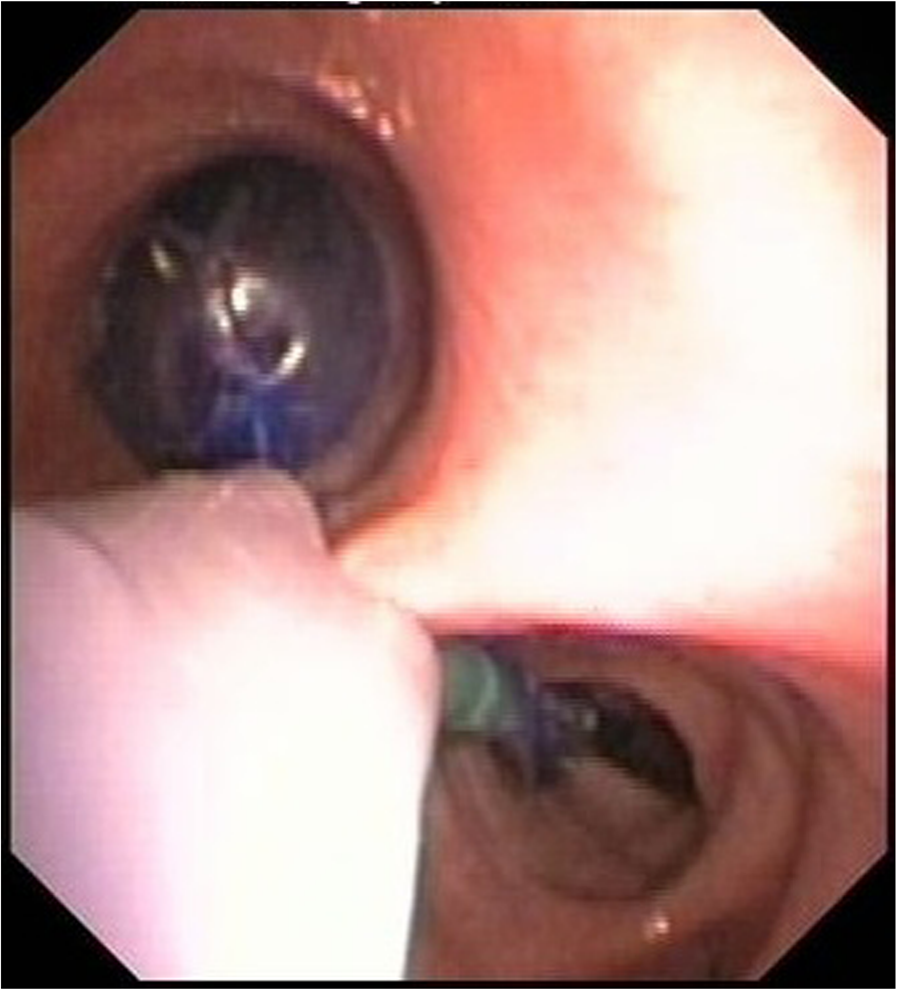
Bronchoscopic view of the EZB with its Y-shaped distal part placed in the right and left mainstem bronchi. The inflated blocker cuff seals the left mainstem bronchus to enable one lung-ventilation for optimal surgical exposure

One-lung ventilation was performed using the EZB (AnaesthetIQ, Rotterdam, The Netherlands). This 7-French, 75-cm, 4-lumina Y-shaped semirigid endobronchial blocker has two different colored distal extensions, both with an inflatable cuff and a small central lumen (Fig. 3). Two pilot balloons at the proximal part of the device serve to inflate/deflate the cuffs. Two additional lumina are available for suction or oxygen insufflation. The EZB is inserted through the designated port on the enclosed multiport adapter. The multiport adapter is designed to connect to an ETT (minimum 7 mm I.D.) and contains two additional upper ports, one for the blocker itself and the other for the bronchoscope. EZB use for lung separation was provided as follows: After the patient had been placed in right decubitus position, the multiport adapter was connected to the NIM EMG ETT. To enable proper deployment of the Y-shaped distal part of the EZB, a minimum of 4 cm distance between the distal end of the NIM EMG ETT and the carina was verified under direct bronchoscopic vision. The EZB was lubricated with silicone spray and introduced through the lockable center port of the multiport adapter with its cuffs completely deflated. Further advance was guided with a fiberoptic bronchoscope (FOB), placing the distal EZB ends into the right and left mainstem bronchi under direct bronchoscopic vision (Fig. 4). If there was less than 4 cm distance between the distal end of the NIM EMG ETT and the carina, the NIM EMG ETT was slightly retracted more proximal. With the EZB finally properly placed, the NIM EMG ETT was readvanced into the trachea as necessary. Both movements required a deflated NIM EMG ETT cuff. To test the bronchial sealing, the cuff of the EZB was inflated with an appropriate volume under direct bronchoscopic vision and deflated again. The insertion technique described was used for all patients and all EZBs were placed under supervision of an attending physician in accordance with the SOP. To facilitate unilateral lung collapse, a specific sequence of action was used after the parietal pleura had been opened by the thoracic surgeon: First, disconnection of the tube from the ventilator allows the operated lung to collapse. After 20 s, reinflation of the blocker cuff under direct bronchoscopic vision with the same volume of air as used before and reconnection of the ETT to the ventilator establishes ventilation of the dependent lung. The adequacy of lung collapse was clinically assessed by the thoracic surgeon. According to our SOP for one-lung ventilation, after removing the endobronchial blocker at the end of surgery, the mucosa of the tracheobronchial system was observed with the FOB for possible damage due to the endobronchial blocker.

Demographic data of the patients, Mallampati score, Cormack and Lehane (CML) classification, surgical characteristics, time-span of clinical experience of the responsible anesthesiologist, anesthesia drugs used for induction and maintenance, airway management, time for EZB placement, problems with EZB placement, clinical adequacy and duration of one-lung ventilation, occurrence and incidence of EZB dislocation or bronchial injury, possible decrease of oxygen saturation, ventilation parameters, need for postoperative ventilation and finally adverse events during surgery were recorded in addition to the described standard monitoring parameters. The data were stored in the electronic patient data management system (NarkoData; IMESO, Hüttenberg, Germany). The acquired data were anonymized and transferred to an Excel datasheet (Microsoft, Redmond, USA) for statistical analysis. Descriptive statistical analysis was done using Statistica version 6 (StatSoft (Europe) GmbH, Hamburg, Germany). Categorical variables were given as absolute numbers and percentages of their occurrence. Continuous variables were presented as medians and interquartile range (IQR).

## Results

A total of 14 patients were enrolled. Demographic and surgical characteristics of the patients are summarized in Table 1. None of the patients displayed any related dysfunction of the RLN preoperatively.

**Table 1:** Demographic and surgical characteristics of the patients

**Patient characteristics**	**n (%) or median (IQR)**
Gender, n (%)	
Female	5 (35.7))
Male	9 (64.3)
Age (y), median (IQR)	59.0 (51.0–68.0)
Weight (kg), median (IQR)	80.5 (66.0–88.0)
Height (cm), median (IQR)	173.0 (158.0–182.0)
BMI (kg/m^2^), median (IQR)	25.7 (22.6–30.0)
ASA physical status, n (%)	
I	0 (0)
II	9 (64.3)
III	5 (35.7)
IV	0 (0)
Mallampati score, n (%)	
I	3 (21.4)
II	7 (50.0)
III	4 (28.6)
IV	0 (0)
CML classification, n (%)	
I	11 (78.6)
II	2 (14.3)
III	0 (0)
IV	0 (0)
not specified	1 (7.1)
**Surgical characteristics**	**n (%) or median (IQR)**
Indications for surgery, n (%)	
Carcinoma of the left upper lobe	9 (64.3)
Left upper lobe metastasis	2 (14.3)
Aortopulmonary window lymph node metastases	3 (21.4)
Surgical procedure, n (%)	
Left upper lobectomy	7 (50.0)
Left upper lobe trisegmentectomy	2 (14.3)
Atypical resection of the left upper lobe	2 (14.3)
Left upper mediastinal lymph node dissection	3 (21.4)
Total operating time (min), median (IQR)	192.5 (176.0–226.0)
Incision/suture time (min), median (IQR)	145.5 (131.0–193.0)
Total anesthesia time (min), median (IQR)	273.5 (243.0–325.0)
Time of one-lung ventilation (min), median (IQR)	92.0 (77.5–132.0)
Time for EZB placement (s), median (IQR)	27.0 (23.0–163.00)

Data are presented as absolute number of patients (%) or as median (IQR)

The involved anesthesiologists had a median clinical experience of 3.5 years (IQR: 3.5–5.3) and worked under close supervision of an attending physician.

The oral intubation with the NIM EMG ETT was uneventful in all patients. Anesthesia induction was performed with etomidate (57%) or propofol (43%). Fentanyl (93%) or remifentanil (7%) were used as intravenous analgetic agents for induction. Rocuronium (93%) or succinylcholine (7%) were used as neuromuscular blocking agents. Repeated doses of rocuronium were necessary in eight patients (57%). However, a significant neuromuscular blockade was excluded by monitoring of the neuromuscular transmission with acceleromyography before the thoracic surgeon tried to stimulate the RLN. Anesthesia was maintained by TIVA using propofol in combination with fentanyl and remifentanil (100%).

The EZB was introduced and positioned without any problems and clinically sufficient lung collapse was achieved in all patients. The median time for EZB placement was 27 s (IQR: 23–163 s). During one-lung ventilation, pressure-controlled ventilation (93%) or volume-controlled-ventilation (7%) was used on the dependent lung. In one patient lung separation had to be interrupted briefly due to severe desaturation (oxygen saturation < 90%). No tracheobronchial injuries or immediate complications from EZB placement or FOB occurred. Thirteen patients (93%) were extubated at the end of surgery in the operation theatre. One patient (7%) required ventilatory support. Postoperatively, all patients were transferred to the intensive care unit.

**Fig. 5:**
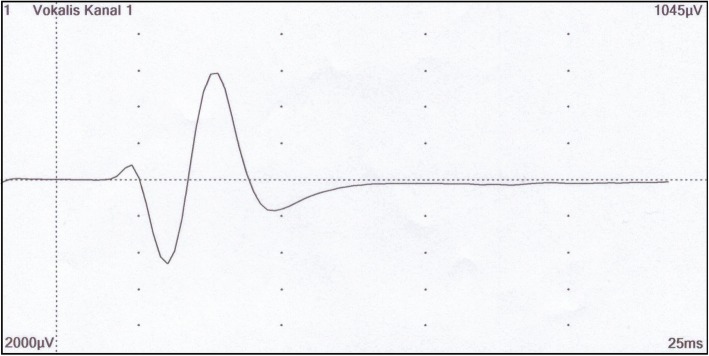
EMG signal of the left RLN recorded intraoperatively

A stable EMG signal was present in all patients (exemplary Fig. 5). No instance of IONM dysfunction occurred. As evidenced by fiberoptic bronchoscopy, no RLN palsy and no negative side effects of the NIM EMG ETT or the EZB were observed postoperatively. However, two of the 14 patients had no control bronchoscopy. One of these patients had to be reintubated due to secondary bleeding the day after surgery. Thus, vocal cord paralysis was excluded by direct laryngoscopy. In the other case no fiberoptic bronchoscopy or laryngoscopy was performed postoperatively. However, an intraoperative EMG response could have been obtained successfully in both cases after dissection and both patients showed no signs of voice disorders after surgery.

## Discussion

RLN injury can result in vocal-cord paresis or paralysis and is associated with a significant morbidity, especially pulmonary complications [7]. In addition, transient or permanent hoarseness is one of the most common causes for medical litigation [11]. Consequently, IONM is frequently used in thyroid surgery to reduce the incidence of RLN injury. Although left upper lobe surgery and left upper mediastinal lymph node dissection are associated with an even higher rate of RLN injury and palsy [3], IONM is not routinely used in these types of operations. Furthermore, data regarding the effectiveness of IONM during thoracic surgery are scarce [8]. Zhao and colleagues described the intraoperative RLN monitoring during thoracotomy as a safe and effective method for identifying the RLN. Unfortunately, the authors did not mention how the intraoperative ventilation was performed [12]. In addition, Kobayashi and colleagues demonstrated that IONM can significantly reduce RLN paralysis in esophageal cancer surgery and is associated with shorter postoperative hospital stay because of a lower rate of postoperative complications [13]. The most common method for IONM is the evoked laryngeal electromyography, which implies the stimulation of the recurrent laryngeal or the vagal nerve and consecutive recording of the EMG signal at the laryngeal muscles via laryngeal needle electromyography, laryngeal surface electrodes or special EMG ETT systems [10]. IONM for thoracic surgical interventions poses a significant challenge because of the need for one-lung ventilation during surgical exposure. Previous studies have reported the use of one or more laryngeal surface EMG electrodes attached to a double-lumen endotracheal tube (DLT) for IONM during single-lung ventilation [4, 14]. However, due to the small dimension of the electrode recording part, maintaining correct positioning of the DLT could be challenging after the patient had been placed in right decubitus position. Thus, reliable laryngeal nerve monitoring and lung separation could interfere. Therefore, we introduced a new method to achieve lung separation and IONM using the EZB in combination with the NIM EMG ETT for patients undergoing thoracic surgery with necessity of one-lung ventilation and high risk for left RLN injury. In the present study the feasibility and effectiveness of this new method were assessed.

No complications were reported, and clinically sufficient lung collapse was achieved in all patients. The EZB was introduced and positioned quickly without any problems and no tracheobronchial injuries occurred. This is in accordance with our previous findings [9]. Vegh and colleagues also described the use of the EZB as safe and easy [15]. However, the use of FOB guidance for initial placement and for repositioning must be considered as mandatory, but this also applies for an electrode-bearing DLT and other BBs. The combination of the EZB and the NIM EMG ETT also provided an identification of the RLN and a stable EMG signal after stimulation of the RLN in all patients. No RLN palsy and no negative side effects of the NIM EMG ETT were observed postoperatively. Thus, the combined use of the EZB and the NIM EMG ETT is a save and easy to apply method to enable reliable IONM and lung separation during left upper lobe surgery and left upper mediastinal lymph node dissection.

Furthermore, in our opinion the described method supersedes the use of self-manufactured combinations of DLT and surface electrodes for IONM during one-lung ventilation. The surface electrodes are not originally designed for the large diameter of a DLT and might detach with the risk of laryngeal damage. Additionally, electrode-bearing DLTs are prone to displacement during patient positioning and surgery. Dislocation of the surface electrodes can cause false absence of nerve signals [4], whereas repositioning of the surface electrodes between the vocal cords may interfere with one-lung ventilation. Although Gelpke and colleagues measured the distance between the true vocal cords and the mainstem bronchi on the preoperative computed tomography (CT) scan to establish the ideal position of the surface electrode on the ETT [4], the distance may differ between supine position (CT scan) and intraoperative right decubitus position (one-lung ventilation). In contrast to an electrode-bearing DLT, the combined use of the NIM EMG ETT and the EZB enables IONM with independent lung separation for optimal surgical exposure. Furthermore, using the NIM EMG ETT and the EZB, IONM is possible even in cases of difficult airway or rapid sequence induction, where electrode-bearing DLTs might not be feasible.

Although IONM of the RLN and one-lung ventilation could also be performed with other BB [16], the main advantage of the EZB compared to other BB is its Y-design, which shows similarities with the anatomic structure of the tracheobronchial tree. The Y-shaped distal part allows the EZB to anchor on the carina and leads to positional stability. The endobronchial blocker is secured between the carina and the seal at the proximal end of the ETT. The two distal extensions, which are positioned in the left and the right mainstem bronchi, mutually stabilize each other by applying counter pressure on the bronchial mucosa in case of surgical manipulation. Thus, the EZB is less prone to secondary malposition than other BBs [17, 18]. Kus and colleagues demonstrated that the EZB had a lower incidence of malpositioning and a shorter time to correct positioning when compared with the Cohen Flex-tip Blocker [18]. In addition, the wire-guided Arndt Endobronchial Blocker is even less stable than the Cohen Flex-Tip Blocker and other BBs [17]. Our previous findings can confirm the positional stability of the EZB [9]. Additionally, the EZB can be positioned quickly and easily [19, 20]. It causes less injury to the tracheal and bronchial mucosa, when compared with the DLT. Furthermore, the positional stability and the quality of lung deflation are equally good [20]. Another advantage of the EZB is the possibility to alternate one-lung ventilation to either lung during bilateral procedures [9]. Despite of the described advantages, there are some limitations that have to be mentioned. First, all surgical approaches involving the mainstem bronchus, such as bronchial sleeve resections or pneumonectomy, exclude the use of any bronchial blocker variant [21]. Second, the speed of lung collapse depends on lung condition. Contamination with secretions or the presence of COPD may delay sufficient lung collapse [22]. Third, the described disconnection technique could not be used for patients being at risk of contaminating the dependent lung with blood or infected secretions. Fourth, another important limitation of the EZB is the small suction channel. The EZB has a 7-French outer diameter, which is split into two lumens leaving a minimal diameter for each lumen. Thus, it is nearly impossible to apply any effective suction to the nondependent lung [23]. However, Yoo and colleagues demonstrated, that the described disconnection technique facilitates equivalent lung collapse when compared with DLT [24]. Fifth, the smallest recommended ETT size for the EZB is 7.0 mm I.D. Thus, the combination of the EZB with the NIM EMG ETT cannot be used for IONM during one-lung ventilation in children. Finally, using the EZB, a minimum of 4 cm distance between the distal end of the NIM EMG ETT and the carina is mandatory to permit the Y-shaped distal part to be deployed properly.

Nevertheless, the combination of the NIM EMG ETT and the EZB provides both reliable IONM and independent lung separation for optimal surgical exposure. Additionally, laryngeal side effects might be reduced in patients receiving an endotracheal intubation with a smaller single-lumen ETT instead of a DLT with attached surface electrodes [25].

Analogous to the monitoring standards in thyroid surgery [10], a reliable and standardised approach to an IONM of the RLN might reduce the high rates of RLN palsy in selected patients undergoing left thoracic surgery. Therefore, we think that the use of the described new method might be beneficial in case of high risk of RLN injury, such as extended lymph node metastases in the aortopulmonary window, left upper lobe surgery after radiotherapy or tumor masses around the left pulmonary artery. Furthermore, IONM might also be beneficial in patients with preexisting palsy of the right RLN. Beyond its use in left thoracic surgery with high risk of RLN injury, there might be possible applications for the described combination of the EZB and the NIM EMG ETT. This could be any surgery requiring dissection next to the intra-thoracic course of the RLN, for example in esophageal surgery. However, further prospective studies in more heterogeneous patient collectives are needed to determine the clinical value of the described method in these applications.

## Conclusions

The described method is technically feasible, easy to apply and save. It enables reliable IONM and provides independent lung separation for optimal surgical exposure during left upper lobe surgery and left upper mediastinal lymph node dissection. The combined use of the EZB and the NIM EMG ETT might propagate the spread of IONM and could reduce the incidence of RLN palsy and impaired lung separation during left thoracic surgery with high risk for RLN injury.

## Data Availability

Please contact corresponding author for data requests.

## Abbreviations

BB: Bronchial blocker
CML: Cormack and Lehane
CT: Computed tomography
DLT: Double-lumen endotracheal tube
EMG: Electromyographic
ETT: Endotracheal tube
EZB: EZ-Blocker
FOB: Fiberoptic bronchoscope
I.D.: Inner diameter
IONM: Intraoperative neuromuscular monitoring
IQR: Interquartile range
RLN: Recurrent laryngeal nerve
SOP: Standard operation procedure
TIVA: Total intravenous anesthesia
